# Adjacent Maxillary Bone Microarchitecture in Patients with Asymmetric Bilateral Lund–Mackay Scores: A Pilot Retrospective Within-Subject Analysis Using CBCT

**DOI:** 10.3390/diagnostics16142270

**Published:** 2026-07-20

**Authors:** Ersen Bilgili, Kaan Orhan

**Affiliations:** 1 Department of Oral and Maxillofacial Radiology, Faculty of Dentistry, Izmir Katip Celebi University, Izmir 35640, Türkiye; 2 Department of Oral and Maxillofacial Radiology, Faculty of Dentistry, Ankara University, Ankara 06500, Türkiye; knorhan@dentistry.ankara.edu.tr; 3 Medical Design Application and Research Center (MEDITAM), Ankara University, Ankara 06590, Türkiye; 4 Department of Oral Radiology, School and Hospital of Stomatology, Cheelo College of Medicine, Shandong University, Jinan 250012, China

**Keywords:** cone-beam computed tomography, maxillary sinusitis, chronic rhinosinusitis, bone remodeling, trabecular bone, Lund–Mackay score

## Abstract

**Background/Objectives**: The study aims to investigate whether lateral asymmetry in the inflammatory burden of the maxillary sinus is associated with measurable differences in adjacent non-alveolar maxillary bone structure, as observed on cone-beam computed tomography (CBCT) images. Additionally, this study aims to examine the relationship between sinus inflammation severity and trabecular bone characteristics using microarchitectural parameters. **Methods**: A total of 100 patients with asymmetric bilateral Lund–Mackay scores were included, yielding 200 maxillary sinuses and exceeding an a priori power requirement of 184 sinuses. Standardized cubic volumes of interest were sampled from the zygomatic process of the maxilla, outside the alveolar process, and analyzed using ImageJ 1.54p and BoneJ+ 0.0.12. Evaluated parameters were trabecular thickness, trabecular separation, bone volume fraction, connectivity, connectivity density, degree of anisotropy, cortical porosity, cortical thickness, and ellipsoid factor. Paired comparisons were performed between the less severe and more severe sinus sides. Generalized estimating equation models were used to assess associations with LM score, age, sex, and etiology of the inflammation as odontogenic or rhinosinusitis. **Results**: Compared to the less severe side, the more severe side showed significantly lower Tb.Th, BV/TV, Cort.Th, and EF and significantly higher Tb.Sp and cortical porosity (all *p* ≤ 0.05). However, Conn., Conn.D, and DA did not differ significantly. An increasing LM score was associated with lower Tb.Th, BV/TV, DA, and EF, as well as higher cortical porosity. Odontogenic and non-odontogenic inflammatory patterns were both associated with less favorable bone characteristics compared to non-inflamed sides. However, the magnitude and distribution of the effects differed across parameters. **Conclusions**: Greater sinus inflammatory burden was associated with measurable alterations in adjacent non-alveolar maxillary bone architecture across multiple morphometric domains. Although not a validated index, the cortical porosity score may reflect the resorptive effects of maxillary sinusitis on the adjacent cortex. The findings support the idea that sinus inflammation may have an osseous correlate beyond the sinus cavity. Further validation in larger, clinically phenotyped cohorts is warranted.

## 1. Introduction

The maxillary sinuses lie in close anatomical continuity with surrounding dentoalveolar and non-alveolar maxillary structures, creating a biologically plausible environment for bidirectional inflammatory influences. Odontogenic maxillary sinusitis is well recognized in oral and maxillofacial radiology, and cone-beam computed tomography (CBCT)-based studies have consistently shown associations between periapical disease, periodontal destruction, implant-related complications, endodontic failure, and Schneiderian membrane thickening or sinus opacification [1,2,3,4,5,6,7,8,9,10,11,12,13,14,15,16]. The recent multidisciplinary literature has also emphasized that accurate diagnosis increasingly depends on coordinated dental and sinonasal assessments supported by dedicated imaging reviews, particularly in unilateral or asymmetric disease presentations [17,18,19].

Odontogenic maxillary sinusitis has been increasingly recognized as a distinct clinical entity with significant effects on the inferior wall of the maxillary sinus. Recent CBCT studies demonstrated that odontogenic lesions can lead to measurable alterations in maxillary bone structure. These findings reinforce the plausibility of sinus-related osseous remodeling beyond the alveolar process [20,21,22]. However, to the best of our knowledge, there is a gap in the literature regarding the possible effect of odontogenic maxillary sinus inflammation on the other walls of the maxillary sinus. Additionally, the possibility that chronic sinus inflammation may influence the adjacent bone architecture has been explored more extensively in rhinology than in dentomaxillofacial imaging. The literature on chronic rhinosinusitis increasingly recognizes osteogenic or neo-osteogenic remodeling as a meaningful component of disease severity, persistence, and postoperative prognosis [23,24,25,26,27,28,29,30,31]. Most previous reports have focused on sinus wall thickening, hyperostosis, or attenuation changes rather than on localized trabecular and cortical characteristics in the adjacent maxillofacial skeleton. Recent studies have characterized neo-osteogenesis and osteitis as clinically meaningful manifestations of recalcitrant maxillary sinus inflammation, with implications for the symptom burden, imaging interpretations, and surgical planning [32,33,34].

CBCT offers a practical in vivo method to investigate this question. Although CBCT cannot replicate the spatial fidelity of micro-computed tomography, several validation studies have shown that standardized CBCT-derived metrics can capture clinically relevant trends in bone macro- and microarchitecture, particularly when the acquisition parameters, segmentation steps, and region selection are carefully controlled [35,36,37,38,39,40]. More recent CBCT studies have shown that voxel size, tube voltage, and acquisition standardization materially influence the accuracy of trabecular and cortical measurements, underscoring the importance of protocol consistency in morphometric research [41,42,43,44]. Therefore, ImageJ- and BoneJ-based workflows have become widely adopted for translational morphometric research on the maxillofacial region [45,46,47].

A major challenge in studying sinus-associated bone changes is the confounding effects of inter-individual variability. Age, sex, skeletal patterns, systemic health, and baseline bone quality may obscure disease-related differences when comparing unrelated individuals. A within-subject paired design partly mitigates this problem by comparing sides in the same patient. For this reason, the present study focused specifically on patients with laterally asymmetric Lund–Mackay (LM) scores.

Accordingly, this study aimed to conduct a pilot investigation to explore the relationship between sinus inflammation asymmetry and the characteristics of the adjacent non-alveolar maxillary bone using CBCT-derived parameters. The primary objective was to compare sinus sides with less and more severe inflammation in the same patients. The secondary objectives were to evaluate observer repeatability and examine whether LM scores, age, sex, and inflammatory etiology are associated with the measured bone parameters.

## 2. Materials and Methods

### 2.1. Study Design and Sample Selection

This retrospective observational CBCT study used archived examinations acquired for various clinical indications at the Faculty of Dentistry of Izmir Katip Celebi University. Ethical approval was obtained prior to data extraction. Sample size estimation was performed using G*Power 3.1.9.7 (University of California, Los Angeles, USA) based on a two-tailed paired comparison with an assumed medium effect size (Cohen’s d = 0.5), α = 0.05, and power = 0.95, indicating a minimum requirement of 184 sinuses. CBCT scans from 100 individuals (200 sinuses) were included in the study.

A total of 823 CBCT examinations were screened using strict inclusion criteria that targeted intraindividual asymmetry. Patients were eligible when both maxillary sinuses could be evaluated and the bilateral LM scores were different. Excluded examinations comprised 212 patients with LM scores of 0 on both sides, 460 with LM scores of 1 on both sides, 48 with LM scores of 2 on both sides, 2 with previous surgery involving the maxillary sinus region, and 1 with fibrous dysplasia affecting the region of interest. Although no cases of silent sinus syndrome were identified, if any had been, they would not have been included in the study. The final sample included approximately 12% of the screened archives.

**Figure 1:**
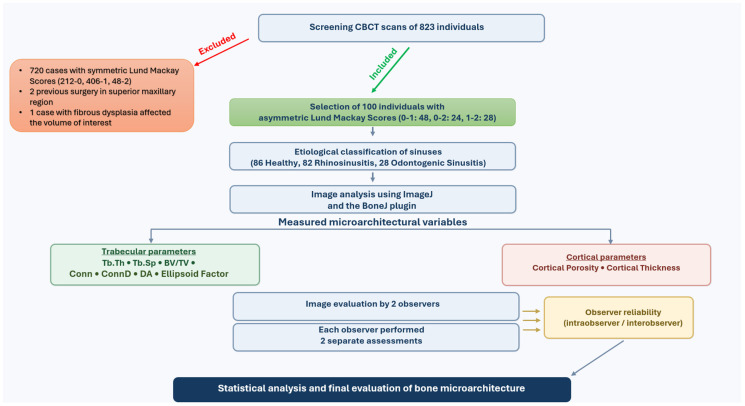
Flow diagram of the study.

The final cohort included 42 women and 58 men, with a mean age of 34.16 ± 17.09 years (range, 14–80 years). The less severe side had an LM score of 0 in 86 patients and 1 in 14 patients, whereas the more severe side had an LM score of 1 in 74 patients and 2 in 26 patients (Figure 1).

### 2.2. Imaging Details and Region-of-Interest Selection

All included scans were acquired in the same institution, by the same technician with a field of view of 15 × 12 cm and a voxel size of 0.2 mm, 5 mA current and 110 kV voltage using NewTom 5G (Cefla SC, Verona, Italy). Supine-positioned patients were instructed to breath though their nose, avoid swallowing, and keep their tongue stuck to their palate with lips slightly closed. The operational threshold for chronic inflammatory involvement was a Schneiderian membrane thickness of at least 4 mm, after which each sinus was graded using the LM system, in which the scoring criteria for each sinus area were as follows: LM = 0, no opacification; LM = 1, partial opacification; and LM = 2, complete opacification [48,49,50,51,52,53,54].

**Figure 2:**
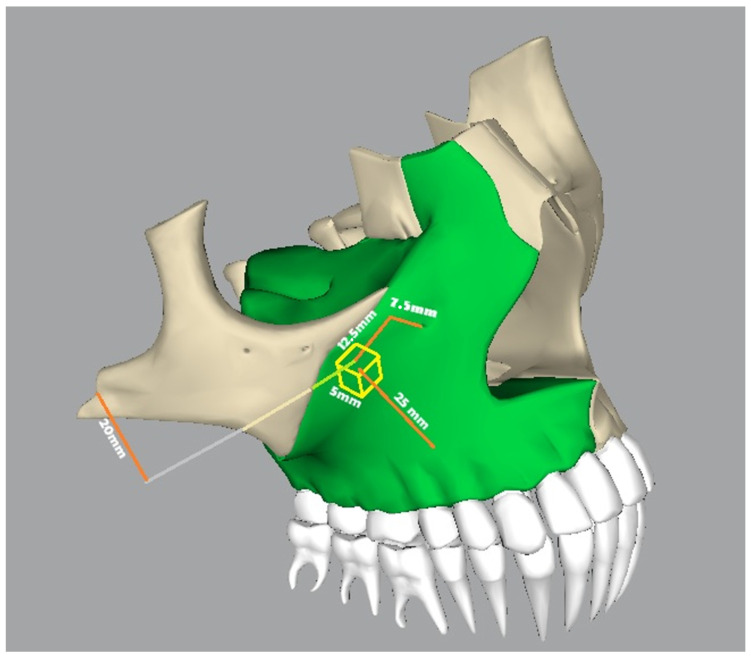
Schematic depiction of volume-of-interest (yellow) selection with distances from anatomical structures (orange).

Recent CBCT studies have demonstrated relationships between mucosal thickening and periapical or periodontal variables, supporting the use of standardized radiological definitions in retrospective sinus research [52,53,54]. Therefore, to reduce the immediate influence of dentoalveolar disease, measurements were performed outside the alveolar process. A standardized region of interest was positioned in the zygomatic process of the maxilla, the center of which was 7.5 mm posterior and 12.5 mm lateral to the infraorbital foramen, 20 mm inferior to the zygomatic arch and 25 mm away from the alveolar region, within trabecular bone adjacent to the sinus-facing cortical plate. Five 5 × 5 mm axial regions of interest were segmented across 25 consecutive slices and stacked to form a 5 mm cubic volume of interest (VOI). The center of the VOI was then placed (Figure 2).

Sequential DICOM slices were imported into ImageJ 1.54p (National Institutes of Health, Bethesda, MD, USA) as calibrated image stacks, and standardized cubic VOIs were generated after anatomical alignment and cropping. A mild Gaussian filter (σ = 2) was applied for noise reduction, followed by stack-based Moments thresholding and binarization. Both examiners performed these steps. Trabecular morphometric parameters were then measured using BoneJ+ 0.0.12 (Department of Imaging Science, University of London, London, UK), which is a plug-in of ImageJ, with the bone represented as the foreground phase for the bone volume fraction (bone volume [BV]/total volume [TV]), trabecular thickness (Tb.Th), and degree of anisotropy (DA); for trabecular separation (Tb.Sp), the binary phase was inverted so that the marrow spaces constituted the foreground. The erosion, dilation, and skeletonization steps were not included in the main workflow because these procedures are not required for BoneJ-based trabecular morphometry and are more closely associated with fractal/skeleton-based analyses [37,44].

The response of bone turnover to metabolic, endocrine, or inflammatory factors is predominantly reflected in trabecular bone, although cortical bone may also demonstrate adaptive changes. In the present study, the trabecular variables Tb.Th, Tb.Sp, BV/TV, connectivity (Conn), Conn density (Conn.D), DA, and ellipsoid factor (EF) were evaluated. Changes in the lateral and posterior cortical walls of the maxillary sinus were also investigated in terms of thickness and integrity using cortical thickness (CortTh) and cortical porosity (CortPor).

**Figure 3:**
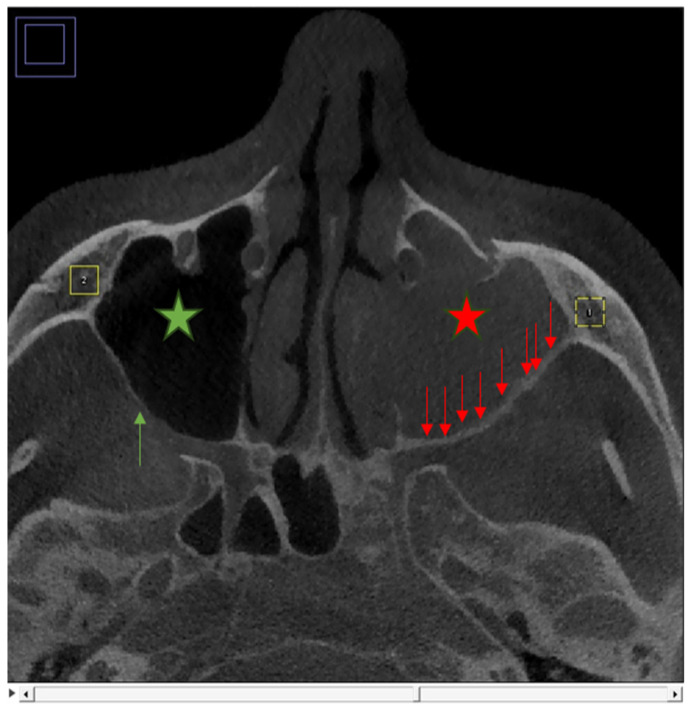
Volume-of-interest selection (1, **right**; 2, **left**) from regions of interest selected in consecutive axial slices (yellow squares). Cortical porosity was scored as 2 (red arrows describe porosities in sinus wall) and 0 (green arrow describes integrity in sinus wall) in sinuses with Lund–Mackay scores of 2 (red star describes complete opacification) and 0 (green star describes no opacification) for the first and second volumes of interest, respectively.

The pilot cortical porosity score (CortPor) for the posterior and lateral walls of the maxillary sinus was developed based on mandibular cortical index scores used for predicting osteoporotic changes in the mandible. The porosity score was defined as: 0 (smooth and intact cortical border), 1 (fewer than three resorptive cavities), and 2 (more than three resorptive cavities) (Figure 3).

The inflammatory etiology was coded as 0 for non-inflammatory, 1 for odontogenic, and 2 for non-odontogenic. This variable was used as an additional explanatory factor in secondary regression models. Etiology was determined based on CBCT findings, including the presence of inflammatory burden in the inferior sinus wall accompanying periapical lesions, periodontal bone loss, or implant-related changes for odontogenic classification, in conjunction with available clinical referral data.

For inferential analysis, the mean of the two observers’ repeated values was used for each side. Intra-observer and inter-observer repeatability were assessed using Dahlberg’s error and intraclass correlation coefficients, and agreement was further assessed using the Bland–Altman methodology [55,56,57].

### 2.3. Statistical Analysis

The principal analytical framework was a paired within-subject comparison between the less severe and more severe sinus sides. Depending on the distribution of the paired differences, either the paired-samples *t*-test or Wilcoxon signed-rank test was used. Descriptive statistics are reported as mean ± standard deviation. All statistical analyses were performed using SPSS (version 21; IBM Corp., Armonk, NY, USA).

To evaluate the factors associated with side-level bone parameters while accounting for clustering within individuals, generalized estimating equation (GEE) models with a Gaussian distribution and exchangeable working correlations were fitted separately for each parameter. The primary model included the LM score, age, and sex. A secondary model replaced the LM score with etiology, while retaining age and sex. All tests were two-sided, and *p*-values < 0.05 were considered statistically significant. Reporting follows the STROBE guidelines for observational studies [58,59,60].

## 3. Results

### 3.1. Sample Overview

Among the 200 evaluated sinuses, LM scores were 0 in 86 sinuses, 1 in 88 sinuses, and 2 in 26 sinuses. The combined etiological distribution was 0 (non-inflamed) in 90 sinuses, 1 (odontogenic) in 28 sinuses, and 2 (non-odontogenic) in 82 sinuses. The reliability analysis demonstrated strong repeatability of the retained study parameters (Table 1). The intra-observer correlation coefficient (ICC1) values ranged from 0.911 to 0.998 for the trabecular and cortical variables included in the final analysis, whereas the inter-observer correlation coefficient (ICC2) values ranged from 0.801 to 0.980.

### 3.2. Reliability Analysis

The detailed intra- and inter-observer repeatability results with Dahlberg’s error values are presented in Table 2. Dahlberg’s error was calculated using the following formula, where d represents the difference between the first and second measurements and n represents the number of paired observations: *DE* = √(Ʃ*d*^2^/2*n*).

**Figure 4:**
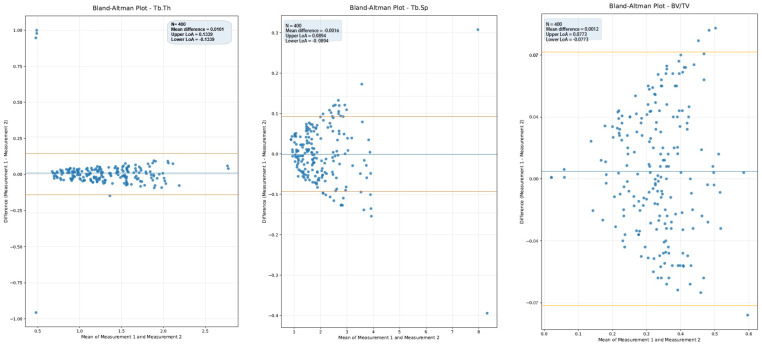
Intra-observer Bland–Altman plots of Tb.Th, Tb.Sp, BV/TV. Orange lines describe the limits of agreement (LoA).

**Figure 5:**
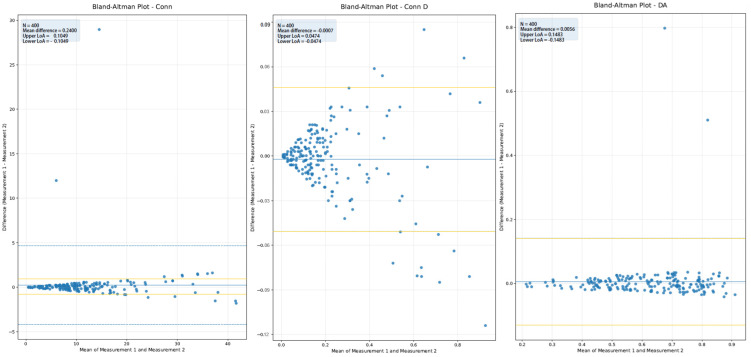
Intra-observer Bland–Altman plots of Conn, Conn.D, DA. Orange lines describe the limits of agreement (LoA).

**Figure 6:**
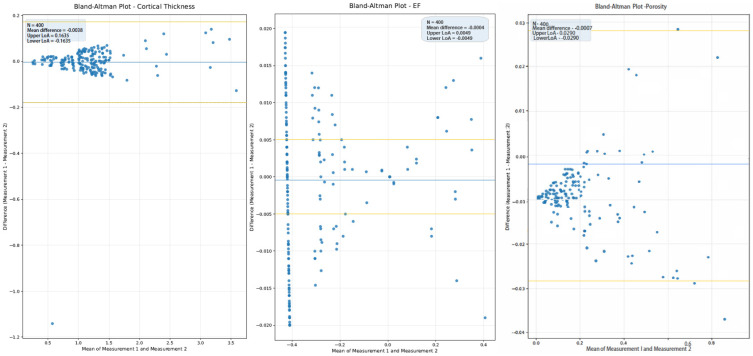
Intra-observer Bland–Altman plots of CortTh, EF, CortPor. Orange lines describe the limits of agreement (LoA).

Cortical porosity, EF, BV/TV, and Conn.D showed particularly strong reliability, supporting the reproducibility of the measurement workflow. Intra-observer Bland–Altman plots are presented in Figure 4, Figure 5 and Figure 6.

Within patients, the more severe side showed significantly lower mean Tb.Th, BV/TV, CortTh, and EF values and significantly higher Tb.Sp and CortPor than the less severe side. Conn, Conn.D, and DA were not significantly different in the paired analysis with the Wilcoxon signed rank test. After Benjamini–Hochberg false discovery rate correction for the nine paired outcomes, significance was retained for Tb.Th, Tb.Sp, BV/TV, CortPor, CortTh, and EF (q ≤ 0.048; Table 3).

In the primary GEE models, a higher LM score was significantly associated with lower Tb.Th, BV/TV, DA, and EF and higher CortPor. The association between Tb.Sp and CortTh was not significant after adjustment. Age was independently associated with several parameters including lower Tb.Th, lower Tb.Sp, higher Conn, higher Conn.D, higher CortPor, higher CortTh, and lower EF. Male sex was positively associated with Conn in the LM-based model (Table 4).

In the secondary models using etiology instead of the LM score, odontogenic inflammation showed strong associations with marked decreases in Tb.Th, BV/TV, Conn, DA, and EF and with increased CortPor. Non-odontogenic inflammation was also associated with deteriorated microarchitectural characteristics, particularly lower Tb.Th, BV/TV, and CortTh and higher Tb.Sp and CortPor relative to the non-inflamed sides (Table 5).

## 4. Discussion

This study was designed as a within-subject pilot analysis to evaluate whether asymmetry in the severity of maxillary sinus inflammation is mirrored by asymmetry in the adjacent non-alveolar maxillary bone. The principal finding was that the more severely inflamed side showed a rarefactive trabecular pattern bone profile, characterized by lower Tb.Th, BV/TV, CortTh, and EF and higher CortPor and Tb.Sp. These findings suggest that the sinus inflammatory burden may be associated with measurable changes not only in mucosal or sinus wall appearance but also in the neighboring maxillary bone microarchitecture.

The paired design is a major strength of this study. By including patients with asymmetric bilateral LM scores, the analysis attempted to minimize confounding introduced by between-subject differences in age, sex, skeletal phenotype, and baseline bone quality. This design is particularly useful in CBCT-based morphometric studies where inter-individual variability can be substantial. At the same time, the reliance on asymmetric LM scores as an inclusion criterion introduced a deliberate enrichment strategy; selection bias may limit the generalizability of the findings, and the results should be interpreted as hypothesis-generating rather than representative of the general population.

The observed reduction in Tb.Th and BV/TV together with increased Tb.Sp supports the interpretation of a structural compromise trabecular framework adjacent to the more severely inflamed sinus side. In the context of chronic rhinosinusitis and osteitis, these findings do not necessarily contradict reports of hyperostosis or sinus wall thickening; rather, they suggest that inflammatory remodeling may be compartment-specific. The sinus wall itself may show thickening or sclerosis, whereas deeper non-alveolar cancellous bone may undergo rarefactive or reorganizational changes. Such compartmental diversity is biologically plausible and aligns with the broader concept of inflammation-related osseous remodeling described in the rhinologic literature [23,24,25,26,27,28,29,30,31,32,33,34].

The cortical findings were particularly noteworthy. The more severely affected side showed higher CortPor and lower CortTh in paired comparisons. In the etiology-adjusted model, non-odontogenic inflammation was associated with lower CortTh, whereas both odontogenic and non-odontogenic inflammation were associated with higher CortPor. These results support the idea that chronic sinus inflammation may be associated with cortical discontinuity or weakening in selected maxillary regions rather than with a uniformly sclerotic phenotype.

The findings using the primary LM-based GEE models further strengthen the main interpretation. Increases in LM scores were independently associated with lower Tb.Th, BV/TV, DA, and EF and with higher CortPor. Although Tb.Sp was higher on the more severe side in the paired comparison, its adjusted association with the LM score was borderline. This pattern is compatible with the known sensitivity of some CBCT-derived morphometric indices to segmentation and resolution while indicating a coherent direction of structural deterioration with increasing inflammatory burden [35,36,37,38,39,40,41,42,43,44].

Etiology-specific modeling provides additional insight into the observed associations. Odontogenic inflammation demonstrated stronger negative associations with Tb.Th, BV/TV, Conn, DA, and EF along with a marked increase in CortPor. Non-odontogenic inflammation also showed adverse associations with Tb.Th, BV/TV, Tb.Sp, and CortPor. Non-odontogenic sinusitis is found to decrease the cortical thickness in maxillary sinus walls while odontogenic sinusitis is found to slightly increase mean cortical thickness. This finding could be because of the sclerosing effect of the increased osteoblastic activity due to inflammation rather than increased health of the tissue. Etiologic differences suggest that the biological route of inflammation influences the pattern of adjacent bone remodeling.

Methodologically, the choice of a 0.2 mm voxel size and a standardized cubic volume of interest in the zygomatic process of the maxilla represents a pragmatic balance between clinical realism and morphometric rigor. CBCT at this resolution cannot be used as a substitute for micro-CT; however, it can be used to detect relative trends when acquisition and processing are standardized. Sampling outside the alveolar process further strengthened the inference that the observed differences were not merely reflections of local periapical or periodontal conditions.

This study had several limitations. First, although within-patient comparisons aimed to avoid systemic factors as much as possible, the retrospective design restricted access to potentially relevant systemic and behavioral variables. Data on smoking status, osteoporosis, sinus hypoventilation, sinus deviation or underwood septa and other local or systemic conditions related to bone metabolism were not available with sufficient reliability for incorporation into the models. These factors may influence the trabecular bone microarchitecture and should be considered in future prospective studies. Further, the strict inclusion criteria targeting intra-individual asymmetry in LM scores may have limited the study’s generalizability. Etiology-specific modeling included odontogenic and non-odontogenic origins (rhinosinusitis); heterogenity in the non-odontogenic group, including allergic rhinitis and polyps, may also be considered a limitation of this study. Moreover, although intra- and inter-observer agreements were generally strong, all image-derived morphometric workflows were dependent on preprocessing and segmentation choices. Apart from these, the pilot CortPor score was adapted conceptually from mandibular cortical index approaches used in osteoporosis screening by the authors; however, the lack of validation remains a limitation. The CortPor score is an ordinal variable; its inclusion in Gaussian GEE models represents an approximation. Although this approach is common in exploratory analyses, ordinal or Poisson GEE models may be more appropriate in future, larger studies. Volumetric investigation of the maxillary sinus is not investigated in the present study, and volumetric changes also may be investigated in future studies.

Patient subgroups also present limitations. For example, the distribution of cases according to LM score was imbalanced, with LM scores of 0, 1 and 2 being represented by 86, 88 and 26 sinuses, respectively. The LM score 2 category was represented by only 26 patients. Although non-parametric tests were performed for evaluation, this remains a limitation. In the etiology-based secondary GEE model, there were also imbalances: 90 healthy sinuses, 82 sinusitis-related sinuses and only 28 odontogenic sinusitis cases. As with the LM score subgroups, this imbalance remains a limitation despite non-parametric tests being performed.

Finally, this study did not link bone findings to treatment outcomes or symptoms; therefore, the clinical prognostic significance of these changes remains unclear.

Despite these limitations, the present findings provide useful radiological information. They suggest that asymmetry in the sinus inflammatory burden may be accompanied by asymmetry in the adjacent maxillary bone microarchitecture and that CBCT-based morphometric analysis can detect this relationship in vivo. Prospective studies with longitudinal follow-up, richer clinical phenotyping, and outcome correlations are needed before these parameters can be considered for routine decision support. Recent reports have also suggested that dental pathology may be under-recognized on sinus CT/CBCT examinations, underscoring the value of coordinated dentomaxillofacial and rhinological interpretation when asymmetric maxillary sinus disease is encountered [22].

## 5. Conclusions

The present pilot investigation demonstrated that the more severely involved side of the maxillary sinus showed less favorable adjacent non-alveolar bone characteristics using CBCT, including lower Tb.Th, BV/TV, CortTh, and EF and higher Tb.Sp and CortPor. Increases in the LM score were independently associated with the deterioration of several bone parameters.

Etiology-specific models suggested that both odontogenic and non-odontogenic inflammatory patterns are linked to different aspects of adjacent skeletal remodeling. These results support the use of this framework as a pilot platform for future prospective studies on sinus-to-bone and bone-to-sinus interactions.

Although not a validated index, the cortical porosity score may reflect the resorptive effects of maxillary sinusitis on the adjacent cortex. Further validation in larger, clinically phenotyped cohorts is warranted.

## Figures and Tables

**Table 1:** Descriptive statistics.

Characteristics	Values
Patients, ***n***	100
Sinuses, ***n***	200
Age, mean ± SD (range), years	34.16 ± 17.09 (14–80)
Sex, ***n***	42 female/58 male
Less severe side LM score 0, ***n***	86
Less severe side LM score 1, ***n***	14
More severe side LM score 1, ***n***	74
More severe side LM score 2, ***n***	26
Etiology 0/1/2, ***n***	90/28/82

**Table 2:** Intra-observer (ICC1) and inter-observer (ICC2) repeatability.

Parameter	N	Dahlberg Error 1	ICC1	Lower 95% LoA 1	Upper 95% LoA 1	Dahlberg Error 2	ICC2	Lower 95% LoA 2	Upper 95% LoA 2
Tb.Th (mm)	400	0.009	0.942	−0.1339	0.1339	0.048	0.910	−0.1756	0.1756
Tb.Sp (mm)	400	0.005	0.990	−0.894	0.894	0.054	0.842	−1.4232	1.4232
BV/TV (%)	400	0.007	0.992	−0.0773	0.0773	0.028	0.925	−0.4540	0.4540
Conn	400	0.010	0.966	−0.1049	0.1049	0.020	0.937	−0.1427	0.1427
Conn.D	400	0.006	0.994	−0.0474	0.0474	0.017	0.928	−0.4740	0.4740
DA	400	0.049	0.911	−0.1483	0.1483	0.054	0.865	−0.1725	0.1725
CortTh (mm)	400	0.010	0.985	−0.1635	0.1635	0.059	0.801	−0.5450	0.5450
EF	400	0.007	0.990	−0.0499	0.0499	0.018	0.943	−0.0876	0.0876
CortPor	400	0.005	0.998	−0.0290	0.0290	0.008	0.980	−0.1450	0.1450

**Table 3:** Paired comparison of less severe and more severe sinus sides with Wilcoxon test.

Parameter	Less Severe Mean ± SD	Less Severe Median (FDR)	More Severe Mean ± SD	More Severe Median (FDR)	Effect Size	*p*	FDR Q *
Tb.Th (mm)	1.443 ± 0.397	1.46 (1.27–1.63)	1.181 ± 0.377	1.28 (1.16–1.38)	0.361	<0.001	<0.001 *
Tb.Sp (mm)	1.927 ± 0.712	1.94 (1.37–2.45)	2.192 ± 1.191	2.39 (1.94–2.93)	0.361	0.032	0.048 *
BV/TV (%)	0.362 ± 0.078	0.35 (0.30–0.41)	0.299 ± 0.098	0.28 (0.24–0.33)	0.361	<0.001	<0.001 *
Conn	12.364 ± 8.457	12.16 (7.55–16.05)	11.027 ± 8.863	11.03 (6.63–16.50)	0.331	0.210	0.237
Conn.D	0.212 ± 0.172	0.21 (0.13–0.25)	0.201 ± 0.203	0.20 (0.11–0.23)	0.361	0.325	0.325
DA	0.639 ± 0.145	0.64 (0.53–0.80)	0.600 ± 0.172	0.60 (0.43–0.71)	0.331	0.060	0.078
CortPor	0.140 ± 0.450	0.14 (0.00–0.36)	0.980 ± 0.791	0.98 (0.45–2.27)	0.361	<0.001	<0.001 *
CortTh (mm)	1.277 ± 0.458	1.28 (1.12–1.41)	1.062 ± 0.568	1.06 (0.79–1.25)	0.361	<0.001	<0.001 *
EF	−0.277 ± 0.223	−0.28 (−0.42–−0.18)	−0.325 ± 0.178	−0.33 (−0.42–−0.28)	0.361	0.005	0.008 *

* q < 0.05 refers to statistical significance.

**Table 4:** Primary GEE models including LM score, age, and sex.

Parameter	LM Score β (*p*) *	Age β (*p*) *	Sex (M/F) β (*p*) *
Tb.Th	−0.206 (<0.001) *	−0.004 (0.040) *	0.050 (0.429)
Tb.Sp	0.165 (0.054)	−0.012 (0.007) *	−0.098 (0.599)
BV/TV	−0.039 (<0.001) *	0.000 (0.596)	0.010 (0.532)
Conn	−0.612 (0.502)	0.126 (0.004) *	2.831 (0.028) *
Conn.D	0.013 (0.472)	0.003 (0.011) *	0.024 (0.424)
DA	−0.062 (<0.001) *	−0.001 (0.393)	−0.024 (0.301)
CortPor	0.624 (<0.001) *	0.012 (<0.001) *	−0.131 (0.138)
CortTh	−0.059 (0.313)	0.007 (0.023) *	0.027 (0.753)
EF	−0.038 (0.001) *	−0.003 (0.002) *	0.007 (0.843)

M, male; F, female; GEE, generalized estimating equation. * *p* < 0.05 refers to statistical significance.

**Table 5:** Secondary GEE models including etiology, age, and sex.

Parameter	Odontogenic β (*p*) *	Non-Odontogenic β (*p*) *	Age β (*p*) *	Sex (M/F) β (*p*) *
Tb.Th	−0.435 (<0.001) *	−0.264 (<0.001) *	−0.003 (0.060)	0.029 (0.631)
Tb.Sp	0.582 (0.093)	0.228 (0.006) *	−0.013 (0.007) *	−0.083 (0.638)
BV/TV	−0.074 (<0.001) *	−0.060 (<0.001) *	0.000 (0.553)	0.006 (0.685)
Conn	−5.060 (<0.001) *	−0.167 (0.892)	0.139 (<0.001) *	2.776 (0.024) *
Conn.D	−0.052 (0.131)	0.007 (0.737)	0.003 (0.007) *	0.026 (0.382)
DA	−0.105 (0.003) *	−0.035 (0.113)	−0.001 (0.414)	−0.033 (0.158)
CortPor	1.018 (<0.001) *	0.835 (<0.001) *	0.012 (<0.001) *	−0.066 (0.425)
CortTh	0.035 (0.786)	−0.190 (0.002) *	0.006 (0.029) *	0.025 (0.764)
EF	−0.070 (<0.001) *	−0.036 (0.073)	−0.003 (0.002) *	0.002 (0.948)

* *p* < 0.05 refers to statistical significance.

## Data Availability

The data that support the findings of this study are available from the corresponding author upon reasonable request.

## Abbreviations

The following abbreviations are used in this manuscript:

CBCT	Cone-Beam Computed Tomography
LM	Lund–Mackay
FDR	False-Discovery Rate
Tb.Th	Trabecular Thickness
Tb.Sp	Trabecular Spacing
BV/TV	Bone Volume/Total Volume (Volume Fraction)
Conn	Connectivity
Conn.D	Connectivity Density
DA	Degree of Anisotropy
CortPor	Cortical Porosity
CortTh	Cortical Thickness
EF	Ellipsoid Factor
GEE	Generalized Estimating Equation
ICC1	Intra-examiner Intraclass Correlation Coefficient
ICC2	Inter-examiner Intraclass Correlation Coefficient
